# The epidemiology of vascular catheter-related bacteraemia outside ICUs in a tertiary care centre

**DOI:** 10.1186/2047-2994-4-S1-O23

**Published:** 2015-06-16

**Authors:** A Hornero, P Saliba, E Shaw, R Escofet, D Garcia, C Ardanuy, F Tubau, M Pujol

**Affiliations:** 1 Infectious Diseases, Bellvitge University Hospital, Hospitalet Llobregat, Spain; 2 Microbiology, Bellvitge University Hospital, Hospitalet Llobregat, Spain

## Introduction

There have been significant changes among non-intensive care unit patients with increasing numbers of comorbidities and invasive procedures, particularly vascular catheterization.

## Objectives

To determine the current epidemiology of vascular catheter-related bacteraemia (CRB) in non-ICU patients over a period of 12 years in a tertiary care hospital.

## Methods

Prospective surveillance of CRB at Bellvitge University Hospital in Barcelona, Spain. CRB was diagnosed through daily meetings between Infection Control Team and microbiologists. For diagnosis of CRB, CDC definitions were used, including presence of phlebitis for peripheral CRB and at least two positive blood cultures for common skin contaminants.

## Results

From January 2003 to December 2014, 561 episodes of CRB were followed. There was a significant reduction in the incidence of CRB from 2003 (0.42 episodes/1.000 patient days) to 2014 (0.20 episodes/1.000 patient days, p<0.000).

Overall, 271 of 651 (42%) episodes were caused by peripheral venous catheter (0.10 ep/1.000 patients-day) and 380 (58%) by central venous catheter (0.14 ep/1.000 patients-day). The most frequent cause of CRB during the study period was short peripheral catheter with 177 episodes (mean days from insertion to bacteraemia: 5d; SD:3d); followed by subclavian with 132 episodes (mean days from insertion to bacteraemia: 18d; SD:18d).

The most frequent causative microorganism were Gram-positive cocci (GPC) (71%) followed by Gram negative bacilli (29%) and fungi (1.2%). Among GPC, 39% were *Staphylococcus aureus* followed by 28% coagulase negative staphylococci. S.aureus caused 50% of short peripheral CRB episodes but only 31% of CVC (p<0.00). Overall mortality was 16.7%, while for patients with *S.aureus* CRB was 26.2% (p<0.00).

## Conclusion

Although there has been a decrease in CRB cases, there remains a significant problem in non-ICU patients. S.aureus is the leading pathogen, particularly among patients with peripheral CRB, and is associated with a high mortality rate.

## Disclosure of interest

None declared.

